# High expression of ubiquitin-conjugating enzyme 2C (UBE2C) correlates with nasopharyngeal carcinoma progression

**DOI:** 10.1186/1471-2407-13-192

**Published:** 2013-04-15

**Authors:** Zhihua Shen, Xiaofan Jiang, Chao Zeng, Shaojiang Zheng, Botao Luo, Yumei Zeng, Ranran Ding, Hanguo Jiang, Qiyi He, Junli Guo, Wei Jie

**Affiliations:** 1 Department of Pathology & Pathophysiology, School of Basic Medicine Science, Guangdong Medical College, Zhanjiang, 524023, People’s Republic of China; 2 Hainan Provincial Key Laboratory of Tropical Medicine, Hainan Medical College, Haikou, 571199, People’s Republic of China; 3 Department of Pathology, People’s Hospital of Zhongshan City, Zhongshan, 528400, People’s Republic of China

**Keywords:** Nasopharyngeal carcinoma, Ubiquitin-conjugating enzyme 2C, Progression, Proliferation, Cell cycle

## Abstract

**Background:**

Overexpression of ubiquitin-conjugating enzyme 2C (UBE2C) has been detected in many types of human cancers, and is correlated with tumor malignancy. However, the role of UBE2C in human nasopharyngeal carcinoma (NPC) is unclear. In this study, we investigated the role of aberrant UBE2C expression in the progression of human NPC.

**Methods:**

Immunohistochemical analysis was performed to detect UBE2C protein in clinical samples of NPC and benign nasopharyngeal tissues, and the association of UBE2C expression with patient clinicopathological characteristics was analyzed. UBEC2 expression profiles were evaluated in cell lines representing varying differentiated stages of NPC and immortalized nasopharyngeal epithelia NP-69 cells using quantitative RT-PCR, western blotting and fluorescent staining. Furthermore, *UBE2C* was knocked down using RNA interference in these cell lines and proliferation and cell cycle distribution was investigated.

**Results:**

Immunohistochemical analysis revealed that UBE2C protein expression levels were higher in NPC tissues than in benign nasopharyngeal tissues (*P*<0.001). Moreover, high UBE2C protein expression was positively correlated with tumor size (*P*=0.017), lymph node metastasis (*P*=0.016) and distant metastasis (*P*=0.015) in NPC patients. *In vitro* experiments demonstrated that UBE2C expression levels were inversely correlated with the degree of differentiation of NPC cell lines, whereas UBE2C displayed low level of expression in NP-69 cells. Knockdown of *UBE2C* led to significant arrest at the S and G2/M phases of the cell cycle, and decreased cell proliferation was observed in poorly-differentiated CNE2Z NPC cells and undifferentiated C666-1 cells, but not in well-differentiated CNE1 and immortalized NP-69 cells.

**Conclusions:**

Our findings suggest that high expression of UBE2C in human NPC is closely related to tumor malignancy, and may be a potential marker for NPC progression.

## Background

Ubiquitination is a crucial molecular mechanism for the degradation of short-lived proteins in eukaryotic cells, and is involved in multiple cellular biological processes including the cell cycle. The process of protein monoubiquitination or polyubiquitination occurs under the control of three types of enzymes: E1 ubiquitin-activating enzymes, E2 ubiquitin-conjugating enzymes and E3 ubiquitin ligase [1]. Human ubiquitin-conjugating enzyme E2C (UBE2C, also called UBCH10) encodes a member of the E2 ubiquitin-conjugating enzyme family [2]. It was reported that UBE2C functions closely with the anaphase-promoting complex/cyclosome (APC/C), which is an E3 ubiquitin ligase that targets cell cycle proteins for degradation by the proteasome [3]. UBE2C is required for the destruction of mitotic cyclins, thereby participating in the regulation of cell cycle progression through M phase [2].

In 2003, Okamoto *et al.* demonstrated that UBE2C expression levels were extremely low in many normal tissues, but prominent in the majority of cancerous cell lines examined, suggesting that UBE2C has the ability to promote cell proliferation and malignant transformation [4]. Recent data has shown that aberrantly high expression of UBE2C contributes to tumorigenesis, and has revealed its potential as a biomarker for cancer prognosis [5]. Abnormally high UBE2C expression was observed in various human solid cancers in the liver [6], thyroid [7], breast [8], colon [9,10], cervix [11], lung [12] and brain [13], and UBE2C expression was positively correlated with invasion depth and tumor node metastasis (TNM) stage in some tumors. Furthermore, inhibition of UBE2C expression induced by RNA interference significantly reduced the proliferation of cancer cells [7,14] and enhanced cell apoptosis *in vitro*[15]. UBE2C transgenic mice are prone to carcinogen-induced lung tumors and a broad spectrum of spontaneous tumors, as UBE2C is a prominent proto-oncogene [16]. Taken together, these data suggest that targeting of UBE2C may be a potential tool for tumor diagnosis and therapy.

Nasopharyngeal carcinoma (NPC) is a type of malignant head and neck cancer derived from the nasopharyngeal epithelium, and is one of the most common malignant diseases in Southern China and Southeast Asia [17]. Almost 85% of NPC patients display a more advanced clinical stage of disease because of the prevalence of lymphadenopathy at first diagnosis [18]. The process of NPC formation and metastasis is complex, and various genes are involved [19] Therefore, it is of great importance to research biomarkers for the early diagnosis, prognosis prediction of NPC and to develop novel therapeutic strategies for NPC. In the present study, we aimed to investigate the role of UBE2C in the progression of NPC. Our results indicated that detection and targeting of UBE2C may be a potentially useful biomarker for NPC treatment.

## Methods

### Patient samples

One hundred and fifteen cases of paraffin-embedded clinical samples were obtained from the Affiliated Hospital of the Guangdong Medical College (Zhanjiang City, Guangdong, China) and the People’s Hospital of Zhongshan City (Zhongshan City, Guangdong, China). In total, 91 cases of NPC (n=91) and 24 cases of nasopharyngeal epithelial hyperplasia (NEH) were examined from 69 men (75.8%) and 22 women (24.2%). Clinical stage was classified based on the pathology tumor–node–metastasis (pTNM) system (AJCC⁄UICC 2002), and all NPC samples were determined to be non-keratinizing carcinoma. NPC patients were diagnosed for the first time at an average age of 42.7 years (range, 23–72 years). Additional clinical data are shown in Table 1. The use of human tissues in this study was approved by the Ethics Council of the Affiliated Hospital of the Guangdong Medical College and the People’s Hospital of Zhongshan City for Approval of Research Involving Human Subjects.

**Table 1:** Clinicopathological characteristics of patient samples and UBE2C expression in NPC

	**N (%)**
**Gender**	
**Male**	**69 (75.8)**
**Female**	**22 (24.2)**
**Age**	
≧**50**	**44 (48.4)**
**< 50**	**47 (51.6)**
**Smoking**	
**Yes**	**42 46.2)**
**No**	**49 (53.8)**
**Clinical classification**	
**I–II**	**15 (16.5)**
**III–IV**	**76 (83.5)**
**T classification**	
**T1–T2**	**31 (34.1)**
**T3–T4**	**60 (65.9)**
**N classification**	
**N0**	**19 (20.9)**
**N1-N3**	**72 79.1)**
**M classification**	
**M0**	**77 (84.6)**
**M1**	**14 (15.4 )**
**Expression of UBE2C**	
**High expression**	**51 (56.0)**
**Low expression**	**40 (44.0)**

### Immunohistochemical analysis of UBE2C protein

The expression and cellular distribution of UBE2C protein was assessed by immunohistochemical analysis. Five micrometer-thick paraffin sections were deparaffinized and re-hydrated according to standard protocols, and heat-induced antigen retrieval was performed in sodium citrate buffer (10 mmol/L, pH6.0). Endogenous peroxidase was inhibited by 0.3% H_2_O_2_, and non-specific protein binding was blocked with 10% goat serum. Sections were then incubated with primary antibody against UBE2C (1:200 dilution; cat. #A-650, Boston Biochem, MA, USA) at 4°C overnight. Non-immune IgG was used as a negative control, and antigenic sites were localized using a SP9000 Polymer Detection System and a 3,3′- diaminobenzidine (DAB) kit (ZSGB-BIO, Beijing, China). The immunoreactive score (IRS) of UBE2C was described previously [20]. Briefly, the staining intensity was determined as 0, negative; 1, weak; 2, moderate; and 3, strong. The percentage of UBE2C-positive cells was scored as 0, no cellular staining; 1, <1% cellular staining; 2, 1–10% cellular staining; 3, 10–33% cellular staining; 4, 33–66% cellular staining; and 5, >66% cellular staining. Samples with a total IRS of <6 were deemed as having low UBE2C expression, and samples with a sum IRS of ≥6 were determined as high UBE2C expression. The scoring of UBE2C was evaluated individually and independently by two pathologists who were double-blinded to the clinical data.

### Cell culture

CNE1, CNE2Z and C666-1 cell lines representing well-, poorly- and undifferentiated NPC, respectively, were grown in Dulbecco’s modified Eagle’s medium (DMEM; Hyclone) supplemented with 10% fetal bovine serum (FBS; Hyclone) and 100 U/ml penicillin and streptomycin (100 μg/ml), as described previously [21]. The immortalized nasopharyngeal epithelial cell line NP-69 (obtained from the lab of Prof. Yao K.T., Cancer Research Institute, Southern Medical University, Guangzhou, China) was cultured in defined keratinocyte serum-free medium (cat. #10744-019, Invitrogen) containing 100 U/ml penicillin, 100 μg/ml streptomycin, 0.2 ng/ml recombinant epidermal growth factor and 5% FBS. All cell lines were cultured at 37°C in a humidified atmosphere with 5% CO_2_.

### RNA interference

siRNAs were purchased from RiboBio Co., Ltd. (Guangzhou, China). For RNA interference (RNAi) experiments, the following double-stranded oligo RNAs specific for the *UBE2C* coding region (si-UBE2C) were used: forward, 5′-GGACACCCAGGGUAACAUAdTdT-3′, reverse, 5′-UAUGUUACCCUGGGUGUCCdTdT-3′. A corresponding scrambled sequence (si-Control, Cat.siB05815) was used as a negative control. One day before transfection, equal numbers of CNE1, CNE2Z, C666-1 and NP-69 cells (5.0×10^5^/ml) were seeded in 6-, 24- and 96-well plates supplemented with complete medium without antibodies. When cells had reached 60–70% confluency, they were transfected with siRNAs using Lipofectamine 2000 (Invitrogen) in Opti-MEM I medium (Invitrogen). Cells were incubated at 37°C in a humidified atmosphere of 5% CO_2_ for 6 h followed by replacement of complete medium. The efficiency of transfection was verified by observation of the fluorescence emitted by the Cy3-conjugated si-Control using fluorescence microscopy.

### Immunofluorescent staining

Indirect immunofluorescence was performed on NPC cells cultured on glass coverslips. After overnight incubation with primary antibody against UBE2C (1/100) at 4°C, the antigenic sites were detected using TRITC-conjugated goat anti-rabbit IgG (1/100, Protein Tech Group, Inc., Chicago, IL, USA). Images of the antigenic sites were captured with a laser scanning confocal microscope (TCS SP5 II; Leica, Germany).

### Western blotting

Total proteins were extracted using RIPA lysis buffer (Cat. # P0013C, Beyotime Institute of Biotechnology, Jiangsu, China). 30 μg total proteins were subjected to SDS-PAGE, and then proteins were transferred to the PVDF membranes. After twice washed with TBST, the membranes were incubated with 5% skimmed milk in TBST at 37°C for 30 min, then the membrane were incubated with the primary antibodies (UBE2C, 1:500, Boston Biochem; β-actin, 1:1000, Santa Cruz, Texas, USA) at 4°C overnight, After twice washed by TBST, the membranes were incubated with horseradish peroxidase (HRP)-conjugated secondary antibodies for 1 hour at 37°C. Bands were visualized using enhanced chemiluminescence (ECL) reagents (Thermo Fisher, Rockford, IL, USA) and analyzed with gel analysis system (BIO-RAD VersDoc TM5000MP System, Guangzhou, China). The expression of β-actin was used as loading control.

### RNA extraction and quantitative RT-PCR

Total RNA was extracted with TaKaRa RNAiso plus reagent (Takara Biotechnology (Dalian) Co., Ltd.). Next, 1 μg of total RNA was used as a template to generate the first strand cDNA by oligo(dT_18_) using the Promega RT System. Pairs of primers (5′–3′) synthesized by Sangon Biotech Co., Ltd. (Shanghai, China) were as follows: *UBE2C* forward: tgatgtctggcgataaagggatt, *UBE2C* reverse: gtgatagcagggcgtgaggaa. β-actin forward, tgacgtggacatccgcaaag, β-actin reverse, ctggaaggtggacagcgagg. PCR was conducted using the LightCycler480 II instrument (Roche (China) Ltd., Shanghai, China). The total reaction volume of 10 μl consisted of 5 μl SYBR Green I PCR Master Mix (Toyobo, Osaka, Japan), 0.4 μl forward primer (10 μM), 0.4 μl reverse primer (10 μM), 1 μl cDNA and 3.2 μl ddH_2_O. The PCR amplification protocol was as follows: denaturation was performed at 95°C for 1 min, followed by 45 PCR cycles of 95°C for 15 s, and 60°C for 60 s. The relative abundance of target mRNAs were determined from the C_T_ values and plotted as the fold change compared with the control group.

### *In vitro* proliferation assays

Proliferation rates were determined by Cell Counting Kit-8 (CCK-8) assays, as described previously [21]. Briefly, 4×10^3^ cells were seeded in 96-well plates at either 24 and 48 h after transfection with or without siRNAs, then 10 μl CCK-8 reagent (Beyotime Institute of Biotechnology, Jiangsu, China) plus 100 μl basal DMEM medium was added per well, and the absorbance of the samples was measured. Each independent experiment was performed three times.

### Cell cycle distribution analysis

NPC cell lines were seeded in 6-well plates and were successfully transfected in triplicate for each set of experimental conditions with the siRNAs described above. Forty-eight hours later, harvested cells were stained with propidium iodide (PI) and subjected to flow cytometric analysis (BD FACSCanto II, MA, USA).

### Statistical analyses

Statistical analyses were carried out using PRISM Software (Version 5. GraphPad Software, CA, USA). Data were analyzed with Chi-square tests and expressed as mean ± SD. For analysis of the differences between two groups, Student’s t-tests were performed. For multiple groups, ANOVA was carried out followed by Student–Newman–Keuls tests. The level of statistical significance was set at *P*<0.05.

## Results

### Immunohistochemical analysis of UBE2C protein expression in NPC and nasopharyngeal tissues

**Figure 1:**
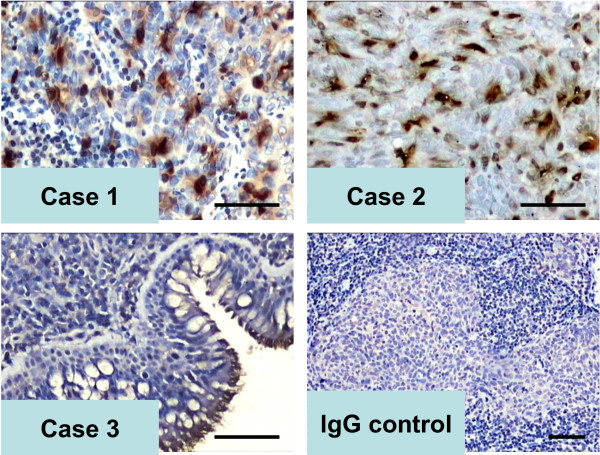
**Representative photographs of high UBE2C expression in NPC samples and low UBE2C expression in non-cancerous nasopharyngeal epithelial hyperplasia (NEH).** The immunohistochemical PV9000 method was used to detect UBE2C protein expression in clinical samples. Non-immune IgG was used as a negative control. The expression and location of UBE2C in cells was revealed by staining with DAB and counterstaining with hematoxylin. IRS >6.0 in NPC (cases 1 & 2) and IRS <6.0 in NEH (case 3). Original magnification for cases 1–3, ×200, for the IgG control, ×100. Scale bar = 100 μM.

First, we investigated the expression of UBE2C in NEH and NPC. Immunohistochemical staining revealed that the majority of NEH cases displayed no or low levels of UBE2C protein expression (IRS <6, 24/24); however, 56% of NPCs (51/91) exhibited strong nuclear and cytoplasmic UBE2C immunoreactivity (IRS ≥6) (*P*<0.001 when compared with NEH), indicating a crucial role of UBE2C expression in the pathogenesis of NPC (Table 1 and Figure 1).

### Relationship between clinicopathological characteristics and UBE2C protein expression in NPC patients

The relationships between clinicopathological parameters and UBE2C protein expression levels in NPCs are detailed in Table 2. There was no significant association of high UBE2C protein expression levels with age, sex, smoking and clinical stage (I–II *vs.* III–IV) in 91 NPC cases. However, we observed that the level of UBE2C protein expression was positively correlated with tumor size (T classification) (T1–T2 *vs*. T3–T4, *P*=0.017), lymph node metastasis (N classification) (N0 vs. N1–N3) (*P*=0.016) and distant metastasis (M classification) (M0 *vs*. M1, *P*=0.015) in NPC patients (Table 2). These data indicated that UBE2C overexpression may be associated with the clinical progression of NPC.

**Table 2:** Correlation between clinicopathological characteristics and UBE2C protein expression in NPC

**Clinical parameters**	**n**	**UBE2C expression**		***χ ***^**2**^	***P*-value**
		**high**	**low**		
**Histological types**					
**NPC**	**91**	**51**	**40**	**26.80**	0.0001*
**NEH**	**24**	**0**	**24**		
**Smoking**					
**Yes**	**42**	**24**	**18**	**0.038**	**0.845**
**No**	**49**	**27**	**22**		
**Gender**					
**Male**	**69**	**37**	**32**	**0.679**	**0.410**
**Female**	**22**	**14**	**8**		
**Age**					
≧**50**	**44**	**22**	**22**	**1.263**	**0.261**
**< 50**	**47**	**29**	**18**		
**Clinical classification**					
**I–II**	**15**	**6**	**9**	**1.877**	**0.171**
**III–IV**	**76**	**45**	**31**		
**T classification**					
**T1–T2**	**31**	**12**	**19**	**5.735**	0.017*
**T3–T4**	**60**	**39**	**21**		
**N classification**					
**N0**	**19**	**6**	**13**	**5.835**	0.016*
**N1-N3**	**72**	**45**	**27**		
**M classification**					
**M0**	**77**	**39**	**38**	**5.913**	0.015*
**M1**	**14**	**12**	**2**		

* Significance as indicated.

### Expression profiles of UBE2C in NPC cell lines *in vitro*

**Figure 2:**
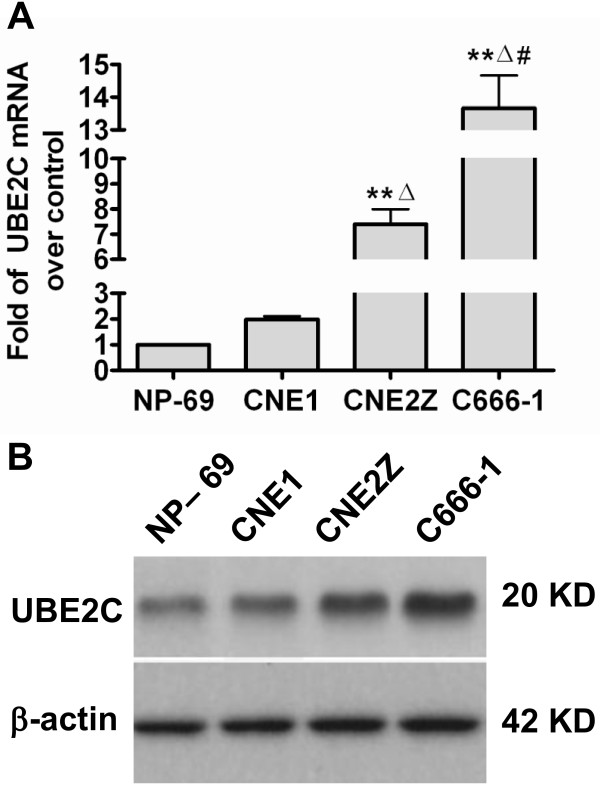
**UBE2C expression in variously differentiated NPC cell lines and NP-69 cells.** (**A**) Well-differentiated CNE1 cells, poorly-differentiated CNE2Z cells, undifferentiated C666-1 cells and immortalized NP-69 cells were seeded in triplicate in 24-well plates for each cell line (n=3), and were harvested for the analysis of *UBE2C* mRNA expression by qRT-PCR; β-actin was used as a loading control. **P<0.01 *vs*. NP-69, ^#^*P*<0.05 *vs.* CNE1, ^Δ^*P*<0.05 *vs.* CNE2Z. (**B**) Western blotting analysis the UBE2C protein expression in CNE1, CNE2Z, C666-1 and NP-69 cells. β-actin was used as a loading control.

**Figure 3:**
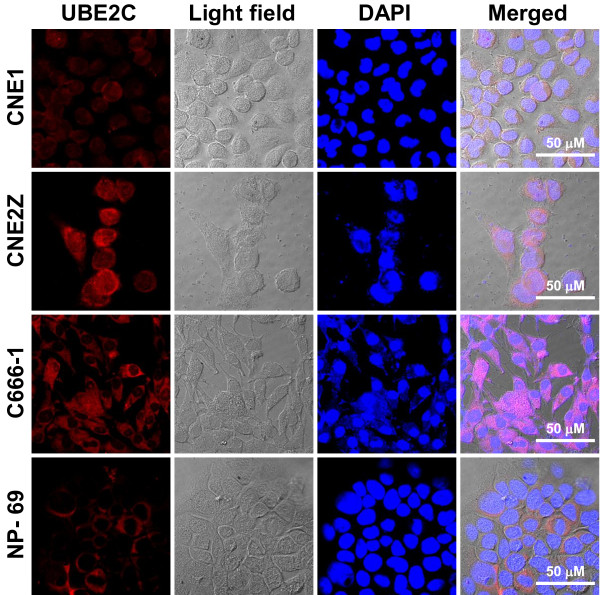
**Representative fluorescent photographs of UBE2C protein expression in various NPC cell lines and NP-69 cells.** Cells grown on glass coverslips were incubated with primary rabbit-anti human UBE2C antibody overnight; the antigenic site of UBE2C was located by TRITC-conjugated goat anti-rabbit IgG (H+L) and photographed by confocal microscopy. Scale bars = 100 μM.

CNE1, CNE-2Z and C666-1 cells were used to further examine the expression profiles of UBE2C in NPC cell lines in the present study. As shown in Figure 2, variable expression of UBE2C was observed at both the mRNA and protein levels in various NPC cell lines. In general, lower expression of UBE2C was detected in highly differentiated CNE1 cells, while increasing expression levels of UBE2C were observed in CNE2Z cells (poorly differentiated NPC) and C666-1 cells (undifferentiated NPC). Low level of UBE2C expression was also observed in immortalized NP-69 cells (Figures 2 and 3). These results indicated that UBE2C was universally expressed in the NPC cell lines, and its expression levels were inversely associated with differentiation status. Finally, immunofluorescent staining showed that UBE2C protein was cytoplasmic in immortalized NP-69 cells, but localized to the cytoplasm and nuclei of NPC cell lines (Figure 3).

### Knockdown of UBE2C attenuates NPC proliferation

**Figure 4:**
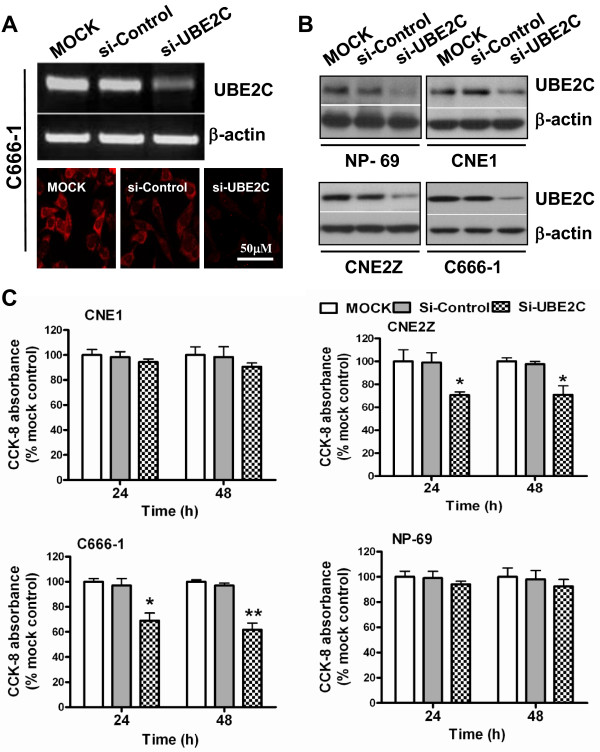
**siRNA inhibited *UBE2C *expression in NPC cells and consequently resulted in attenuated cell proliferation.** (**A**) C666-1 cells were transfected with si-UBE2C or si-Control or without siRNA (MOCK). Forty-eight hours later, *UBE2C* expression was assessed by PCR and immunofluorescence using TRITC-conjugated IgG (H+L). (**B**) Western blotting analysis the UBE2C protein expression in various cell lines post tranfection of siRNAs, the β-actin was used as loading controls. (**C**) Twenty-four and 48 h after transfection, CCK-8 assays were used to analyze the proliferation of various types of NPC cells. Values of optical density (OD) were obtained by the absorbance at the dual wavelengths 450/630 nM, and the results indicating the cell viability were plotted as the percentage over controls (MOCK cells). * *P*<0.05, ** *P*<0.01 *vs*. mock or si-Control–treated groups.

Forced UBE2C expression in NIH 3T3 cells has been shown to promote cell proliferation [4]. Thus, we examined the role of UBE2C in NPC cell proliferation. Three pairs of RNA oligos targeting different regions of the *UBE2C* gene coding region were designed to knockdown UBE2C expression (data not shown). We found that the double-stranded oligos targeting the sequence GGACACCCAGGGTAACATA (401–420nt of CDS of *UBE2C*) displayed the most powerful inhibitory effects (>75%). As shown in Figure 4A, si-UBE2C attenuated UBE2C expression both at the mRNA and protein levels in high UBE2C-expressing C666-1 cells, indicating these siRNA oligos function well. Therefore, these double-stranded RNA oligos were used in the subsequent experiments. And the results of western blotting further confirmed that transfection this siRNAs to NPC cells led to a significant decrease of UBE2C protein expression (Figure 4B). Then the cell proliferation was examined by CCK-8 assays post transfection these 4 cell line with UBE2C specific siRNA. As shown in Figure 4C, transfection of the NPC cell lines with this siRNA led to significantly damaged cell viability in CNE2Z and C666-1 cells, but not the CNE1 and NP-69 cells. Together, these results suggest that overexpression of UBE2C plays a crucial role in NPC cell proliferation.

### Knockdown of UBE2C arrests NPC cells at S and G2/M phases

**Figure 5:**
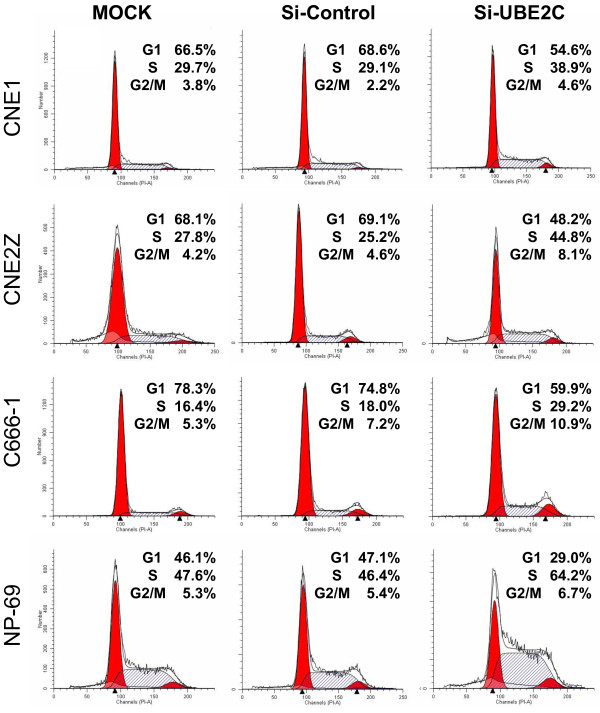
**Knockdown of *UBE2C *with siRNA arrested the cell cycle at G2-M and S phase.** CNE1, CNE2Z, C666-1 and NP-69 cells were transfected with *UBE2C* siRNA (si-UBE2C) or si-Control or left untransfected (MOCK) for 48 h, then the cell cycle was assessed by FCM.

UBE2C is involved in many points of cell cycle control [5]. In the present study, treatment of the NPC cell lines with si-UBE2C decreased the distribution of cells in G1 phase but increased the proportion in S and G2/M phase. As shown in Figure 5, the increases in the proportion of NP-69, CNE1, CNE2Z and C666-1 cells in S phase was 35.7%, 30.9%, 79.9% and 141.6%, respectively. Furthermore, the increase in the proportion of NP-69, CNE1, CNE2Z and C666-1 cells in G2/M phase was 26.4%, 21.1% 92.8% and 110.3%, respectively. These results suggested that inhibition of UBE2C expression in UBE2C highly-expressing NPC cells led to a significant re-distribution in the cell cycle.

## Discussion

In the present study, we first found that UBE2C was predominantly expressed in NPC samples, whereas it was weakly expressed in nasopharyngeal tissues; moreover, we found that high UBE2C protein expression was positively related to tumor size, lymph node metastasis and distant metastasis in NPC patients. These results indicated that high expression of UBE2C was closely related to the clinical progression of NPC. Consequently, we examined UBE2C expression in variously differentiated NPC cell lines *in vitro.* The results showed that immortalized nasopharyngeal NP-69 cells displayed low level of UBE2C expression; however, UBE2C was universally expressed in a variety of NPC cell lines, and its expression levels were reversely related to the stages of differentiation. Finally, treatment of the NPC cells with *UBE2C*-specific siRNA led to a decrease in cell proliferation and arrest at S and G2/M phase of the cell cycle, suggesting that targeting of UBE2C is a potential anti-NPC therapeutic strategy. To the best of our knowledge, this is the first report regarding the relation of aberrant expression of UBE2C with NPC malignancy.

Human UBE2C belongs to the E2 ubiquitin-conjugating enzyme family [2], which functions closely with APC/C [3]. Expression of UBE2C is required for the destruction of mitotic cyclins, for example cyclin B, to promote cell cycle progression from M to G1 phase [2]. Therefore, overexpression of UBE2C contributes to increased cell proliferation, and as a result, cancer cells acquire a hallmark of tumorigenicity through uncontrolled cell proliferation. Early work by Fang *et al.* revealed that some candidate biomarkers for cancer, including UBE2C, were upregulated in NPC [22]. In the present study, we found that high expression of UBE2C protein was detected in 56.0% NPC cases, whilst no UBE2C expression was observed in benign nasopharyngeal tissues; moreover, high UBE2C expression was found to be positively associated with the T, M and N classifications of NPC, indicating that high expression of UBE2C contributes to the pathogenesis and clinical progression of NPC, although these findings require further validation in larger cohorts. Our results were consistent with other reports describing overexpression of UBE2C in many types of tumors, and demonstrate that detection of UBE2C may be a potential biomarker for tumor diagnosis or prognostic judgment [6-9,13,20,23-29].

By using a variety of differentiated stages of NPC cell lines, the UBE2C expression profiles were further analyzed. Well-differentiated CNE1, poorly-differentiated CNE2Z and undifferentiated C666-1 cells used in the present investigation were representative of NPC. We found that when compared with the immortalized NP-69 cells, UBE2C mRNA and protein were universally expressed in these NPC cell lines. Generally, UBE2C expression was found to be inversely related with the differentiation stages of NPC cells. Poor differentiation in cancer cells implies a higher degree of malignancy, and as a hallmark of tumorigenesis, upregulated cell proliferation and migration was acquired. As a result, after treatment of the NPC cell lines with UBE2C-specific siRNA, attenuated cell proliferation was observed. Our results revealed that targeting UBE2C in NPC cells may be beneficial for NPC molecular treatment. These *in vitro* results were also consistent with other reports that targeting UBE2C may be a useful therapeutic strategy in various cancers, such as cervical, colorectal and esophageal carcinomas [11,14,25,30,31].

Cell cycle progression is precisely mediated by a combination of cyclin-dependent kinases, kinase inhibitors and protein phosphorylation. The timely and specific degradation of cyclins and kinase inhibitors at critical check points in the cell cycle by the ubiquitin-proteasome system (UPS) also participates in this process. The cell-cycle G_2_-M phase gene *UBE2C* encompasses the cell cycle window associated with exit from mitosis. Depletion of UBE2C in cancer cells by *UBE2C*-siRNA redistributes the cell cycle phases [14,25], while bortezomib or cell-cycle inhibitor-779 (CCI-779) stabilizes mitotic cyclins and prevents cell cycle progression via attenuation of UBE2C transcription and mRNA stability [30,32]. Our present results revealed that knockdown of *UBE2C* in NPC cells caused significant cell-cycle G2-M and S accumulation. As our results show, transfection of the most highly UBE2C-expressing C666-1 cells with siRNA for 48 h lead to a 141.6% increase in G2-M and 110.3% increase in S phase, implying a crucial role of UBE2C in NPC cell cycle determination. Our results support the findings of Lin *et al*., who reported that inhibition of UBE2C in Seg-1 cells with si-UBE2C resulted in the re-distribution of the cell cycle [25].

The *UBE2C* gene is localized to 20q13.1, a chromosomal region frequently associated with genomic amplification in many types of cancers. It was reported that genomic amplification was a mechanism of increased UBE2C expression in colon cancer, thyroid carcinoma and prostate cancer [23,33,34]. Extensive chromosomal copy number aberrations were also observed in NPC [35,36]. High frequencies of allelic imbalances at chromosomes 3p, 9p, 11q, 12q, 13q, 14q, and 16q were detected in primary NPC [37]. Very recently, Hu *et al.* reported a series of chromosomal abnormalities, including some of those hot spots mentioned above, in C666-1 cells and NPC biopsies [38]. In contrast to the previous investigations regarding amplification of 20q in some human tumors [23,33,34], the loss of 20q in NPC was reported by Yan *et al.*[39]. We did not examine the amplification of 20q in the present study; thus, the mechanism of high expression of UBE2C in NPC requires further elucidation.

NPC is an Epstein Barr virus (EBV) associated malignant carcinoma. The EBV- positive NPC cells display much aggressiveness, which has been reported previously by various labs. It was reported that in papillomavirus type 16 E6- and E7- expressing keratinocytes, a high expression of UBE2C was observed, which may lead to the bypass of the spindle assembly checkpoint even with the DNA injury [40]. In NPC cells, EBV may impair cell cycle checkpoint via its encoded lament membrane protein [41]. Thus, the possible relationship between the infection of EBV and up-regulation of UBE2C in NPC should deserver much attention.

## Conclusions

We provided the first evidence that high UBE2C expression is closely related to the clinical progression of NPC. UBE2C was universally expressed in all NPC cell lines examined, and its expression levels were inversely related with cell differentiation; knockdown of UBE2C by specific siRNA led to attenuated cell proliferation and cell cycle arrest at G_2_-M and S phases. Our results indicated that detection and targeting of UBE2C may be beneficial for NPC treatment.

## Competing interests

The authors declare no competing interests.

## Authors’ contributions

ZS participated in the design of the study, performed statistical analysis and drafted the manuscript. XJ, ZC, RD performed the experiments. SZ, QH participated in the design of the study and helped to draft the manuscript. YZ collected the clinical samples and participated in the design of the study. BL, HJ collected the clinical samples and scored the immunohistochemistry. JG, WJ conceived and coordinated the study. All the authors read and approved the final manuscript.

## Pre-publication history

The pre-publication history for this paper can be accessed here:

http://www.biomedcentral.com/1471-2407/13/192/prepub

## References

[B1] Hershko A Ciechanover A The ubiquitin system Annu Rev Biochem 1998 67 425 479 10.1146/annurev.biochem.67.1.425 9759494

[B2] Townsley FM Aristarkhov A Beck S Hershko A Ruderman JV Dominant-negative cyclin-selective ubiquitin carrier protein E2-C/UbcH10 blocks cells in metaphase Proc Natl Acad Sci U S A 1997 94 2362 2367 10.1073/pnas.94.6.2362 9122200 PMC20093

[B3] Lin Y Hwang WC Basavappa R Structural and functional analysis of the human mitotic-specific ubiquitin-conjugating enzyme, UbcH10 J Biol Chem 2002 277 21913 21921 10.1074/jbc.M109398200 11927573

[B4] Okamoto Y Ozaki T Miyazaki K Aoyama M Miyazaki M Nakagawara A UbcH10 is the cancer-related E2 ubiquitin-conjugating enzyme Cancer Res 2003 63 4167 4173 12874022

[B5] Hao Z Zhang H Cowell J Ubiquitin-conjugating enzyme UBE2C: molecular biology, role in tumorigenesis, and potential as a biomarker Tumour Biol 2012 33 723 730 10.1007/s13277-011-0291-1 22170434

[B6] Ieta K Ojima E Tanaka F Nakamura Y Haraguchi N Mimori K Inoue H Kuwano H Mori M Identification of overexpressed genes in hepatocellular carcinoma, with special reference to ubiquitin-conjugating enzyme E2C gene expression Int J Cancer 2007 121 33 38 10.1002/ijc.22605 17354233

[B7] Pallante P Berlingieri MT Troncone G Kruhoffer M Orntoft TF Viglietto G Caleo A Migliaccio I Decaussin-Petrucci M Santoro M Palombini L Fusco A UbcH10 overexpression may represent a marker of anaplastic thyroid carcinomas Br J Cancer 2005 93 464 471 10.1038/sj.bjc.6602721 16106252 PMC2361574

[B8] Berlingieri MT Pallante P Sboner A Barbareschi M Bianco M Ferraro A Mansueto G Borbone E Guerriero E Troncone G Fusco A UbcH10 is overexpressed in malignant breast carcinomas Eur J Cancer 2007 43 2729 2735 10.1016/j.ejca.2007.09.003 17933517

[B9] Fujita T Ikeda H Taira N Hatoh S Naito M Doihara H Overexpression of UbcH10 alternates the cell cycle profile and accelerate the tumor proliferation in colon cancer BMC Cancer 2009 9 87 10.1186/1471-2407-9-87 19302711 PMC2666760

[B10] Chen S Chen Y Hu C Jing H Cao Y Liu X Association of clinicopathological features with UbcH10 expression in colorectal cancer J Cancer Res Clin Oncol 2010 136 419 426 10.1007/s00432-009-0672-7 19779934 PMC11827792

[B11] Bose MV Gopal G Selvaluxmy G Rajkumar T Dominant negative Ubiquitin-conjugating enzyme E2C sensitizes cervical cancer cells to radiation Int J Radiat Biol 2012 88 629 634 10.3109/09553002.2012.702299 22694363

[B12] Perrotta I Bruno L Maltese L Russo E Donato A Donato G Immunohistochemical analysis of the ubiquitin-conjugating enzyme UbcH10 in lung cancer: a useful tool for diagnosis and therapy J Histochem Cytochem 2012 60 359 365 10.1369/0022155412439717 22388643 PMC3351232

[B13] Jiang L Huang CG Lu YC Luo C Hu GH Liu HM Chen JX Han HX Expression of ubiquitin-conjugating enzyme E2C/UbcH10 in astrocytic tumors Brain Res 2008 1201 161 166 18331723 10.1016/j.brainres.2008.01.037

[B14] Chen SM Jiang CY Wu JY Liu B Chen YJ Hu CJ Liu XX RNA interference-mediated silencing of UBCH10 gene inhibits colorectal cancer cell growth in vitro and in vivo Clin Exp Pharmacol Physiol 2010 37 525 529 10.1111/j.1440-1681.2010.05348.x 20529090

[B15] Jiang L Bao Y Luo C Hu G Huang C Ding X Sun K Lu Y Knockdown of ubiquitin-conjugating enzyme E2C/UbcH10 expression by RNA interference inhibits glioma cell proliferation and enhances cell apoptosis in vitro J Cancer Res Clin Oncol 2010 136 211 217 10.1007/s00432-009-0651-z 19657671 PMC11828233

[B16] van Ree JH Jeganathan KB Malureanu L van Deursen JM Overexpression of the E2 ubiquitin-conjugating enzyme UbcH10 causes chromosome missegregation and tumor formation J Cell Biol 2010 188 83 100 10.1083/jcb.200906147 20065091 PMC2812857

[B17] Cao SM Simons MJ Qian CN The prevalence and prevention of nasopharyngeal carcinoma in China Chin J Cancer 2011 30 114 119 10.5732/cjc.010.10377 21272443 PMC4013340

[B18] Ho FC Tham IW Earnest A Lee KM Lu JJ Patterns of regional lymph node metastasis of nasopharyngeal carcinoma: a meta-analysis of clinical evidence BMC Cancer 2012 12 98 10.1186/1471-2407-12-98 22433671 PMC3353248

[B19] Cho WC Nasopharyngeal carcinoma: molecular biomarker discovery and progress Mol Cancer 2007 6 1 17199893 10.1186/1476-4598-6-1 PMC1774581

[B20] Donato G Iofrida G Lavano A Volpentesta G Signorelli F Pallante PL Berlingieri MT Pierantoni MG Palmieri D Conforti F Maltese L Tucci L Amorosi A Fusco A Analysis of UbcH10 expression represents a useful tool for the diagnosis and therapy of astrocytic tumors Clin Neuropathol 2008 27 219 223 18666437 10.5414/npp27219

[B21] Jie W He QY Luo BT Zheng SJ Kong YQ Jiang HG Li RJ Guo JL Shen ZH Inhibition of Pim-1 attenuates the proliferation and migration in nasopharyngeal carcinoma cells Asian Pac J Trop Med 2012 5 645 650 10.1016/S1995-7645(12)60132-1 22840454

[B22] Fang W Li X Jiang Q Liu Z Yang H Wang S Xie S Liu Q Liu T Huang J Xie W Li Z Zhao Y Wang E Marincola FM Yao K Transcriptional patterns, biomarkers and pathways characterizing nasopharyngeal carcinoma of Southern China J Transl Med 2008 6 32 10.1186/1479-5876-6-32 18570662 PMC2443113

[B23] Takahashi Y Ishii Y Nishida Y Ikarashi M Nagata T Nakamura T Yamamori S Asai S Detection of aberrations of ubiquitin-conjugating enzyme E2C gene (UBE2C) in advanced colon cancer with liver metastases by DNA microarray and two-color FISH Cancer Genet Cytogenet 2006 168 30 35 10.1016/j.cancergencyto.2005.12.011 16772118

[B24] Berlingieri MT Pallante P Guida M Nappi C Masciullo V Scambia G Ferraro A Leone V Sboner A Barbareschi M Ferro A Troncone G Fusco A UbcH10 expression may be a useful tool in the prognosis of ovarian carcinomas Oncogene 2007 26 2136 2140 10.1038/sj.onc.1210010 17016443

[B25] Lin J Raoof DA Wang Z Lin MY Thomas DG Greenson JK Giordano TJ Orringer MB Chang AC Beer DG Lin L Expression and effect of inhibition of the ubiquitin-conjugating enzyme E2C on esophageal adenocarcinoma Neoplasia 2006 8 1062 1071 10.1593/neo.05832 17217624 PMC1783715

[B26] Rajkumar T Sabitha K Vijayalakshmi N Shirley S Bose MV Gopal G Selvaluxmy G Identification and validation of genes involved in cervical tumourigenesis BMC Cancer 2011 11 80 10.1186/1471-2407-11-80 21338529 PMC3050856

[B27] Troncone G Guerriero E Pallante P Berlingieri MT Ferraro A Del Vecchio L Gorrese M Mariotti E Iaccarino A Palmieri EA Zeppa P Palombini L Fusco A UbcH10 expression in human lymphomas Histopathology 2009 54 731 740 10.1111/j.1365-2559.2009.03296.x 19438748

[B28] Psyrri A Kalogeras KT Kronenwett R Wirtz RM Batistatou A Bournakis E Timotheadou E Gogas H Aravantinos G Christodoulou C Makatsoris T Linardou H Pectasides D Pavlidis N Economopoulos T Fountzilas G Prognostic significance of UBE2C mRNA expression in high-risk early breast cancer. A Hellenic Cooperative Oncology Group (HeCOG) study Ann Oncol 2012 23 1422 1427 10.1093/annonc/mdr527 22056852

[B29] Vasiljevic A Champier J Figarella-Branger D Wierinckx A Jouvet A Fevre-Montange M Molecular characterization of central neurocytomas: potential markers for tumor typing and progression Neuropathology 2013 33 149 161 10.1111/j.1440-1789.2012.01338.x 22816789

[B30] Bavi P Uddin S Ahmed M Jehan Z Bu R Abubaker J Sultana M Al-Sanea N Abduljabbar A Ashari LH Alhomoud S Al-Dayel F Prabhakaran S Hussain AR Al-Kuraya KS Bortezomib stabilizes mitotic cyclins and prevents cell cycle progression via inhibition of UBE2C in colorectal carcinoma Am J Pathol 2011 178 2109 2120 10.1016/j.ajpath.2011.01.034 21514426 PMC3081207

[B31] Wagner KW Sapinoso LM El-Rifai W Frierson HF Butz N Mestan J Hofmann F Deveraux QL Hampton GM Overexpression, genomic amplification and therapeutic potential of inhibiting the UbcH10 ubiquitin conjugase in human carcinomas of diverse anatomic origin Oncogene 2004 23 6621 6629 10.1038/sj.onc.1207861 15208666

[B32] Wang H Zhang C Rorick A Wu D Chiu M Thomas-Ahner J Chen Z Chen H Clinton SK Chan KK Wang Q CCI-779 inhibits cell-cycle G2-M progression and invasion of castration-resistant prostate cancer via attenuation of UBE2C transcription and mRNA stability Cancer Res 2011 71 4866 4876 10.1158/0008-5472.CAN-10-4576 21593191 PMC3138908

[B33] Lee JJ Au AY Foukakis T Barbaro M Kiss N Clifton-Bligh R Staaf J Borg A Delbridge L Robinson BG Wallin G Hoog A Larsson C Array-CGH identifies cyclin D1 and UBCH10 amplicons in anaplastic thyroid carcinoma Endocr Relat Cancer 2008 15 801 815 10.1677/ERC-08-0018 18753363

[B34] Tzelepi V Zhang J Lu JF Kleb B Wu G Wan X Hoang A Efstathiou E Sircar K Navone NM Troncoso P Liang S Logothetis CJ Maity SN Aparicio AM Modeling a lethal prostate cancer variant with small-cell carcinoma features Clin Cancer Res 2012 18 666 677 10.1158/1078-0432.CCR-11-1867 22156612 PMC3923417

[B35] Lo KW Chung GT To KF Deciphering the molecular genetic basis of NPC through molecular, cytogenetic, and epigenetic approaches Semin Cancer Biol 2012 22 79 86 10.1016/j.semcancer.2011.12.011 22245473

[B36] Li X Wang E Zhao YD Ren JQ Jin P Yao KT Marincola FM Chromosomal imbalances in nasopharyngeal carcinoma: a meta-analysis of comparative genomic hybridization results J Transl Med 2006 4 4 10.1186/1479-5876-4-4 16423296 PMC1403800

[B37] Lo KW Teo PM Hui AB To KF Tsang YS Chan SY Mak KF Lee JC Huang DP High resolution allelotype of microdissected primary nasopharyngeal carcinoma Cancer Res 2000 60 3348 3353 10910036

[B38] Hu C Wei W Chen X Woodman CB Yao Y Nicholls JM Joab I Sihota SK Shao JY Derkaoui KD Amari A Maloney SL Bell AI Murray PG Dawson CW Young LS Arrand JR A global view of the oncogenic landscape in nasopharyngeal carcinoma: an integrated analysis at the genetic and expression levels PLoS One 2012 7 e41055 10.1371/journal.pone.0041055 22815911 PMC3398876

[B39] Yan W Song L Wei W Li A Liu J Fang Y Chromosomal abnormalities associated with neck nodal metastasis in nasopharyngeal carcinoma Tumour Biol 2005 26 306 312 10.1159/000089289 16254460

[B40] Patle D McCance DJ Compromised spindle assembly checkpoint due to altered expression of Ubch10 and Cdc20 in human papillomavirus type 16 E6- and E7-expressing keratinocytes Virol 2010 84 10956 10964 10.1128/JVI.00259-10 PMC2953194 20739533

[B41] Deng W Pang PS Tsang CM Hau PM Yip YL Cheung AL Tsao SW Epstein-Barr virus-encoded latent membrane protein 1 impairs G2 checkpoint in human nasopharyngeal epithelial cells through defective Chk1 activation PLoS One 2012 7 e39095 10.1371/journal.pone.0039095 22761726 PMC3382577

